# The role of water molecules in stereoselectivity of glucose/galactose-binding protein

**DOI:** 10.1038/srep36807

**Published:** 2016-11-09

**Authors:** Minsup Kim, Art E. Cho

**Affiliations:** 1 Department of Bioinformatics, Korea University, 2511 Sejong-ro, Sejong 339-700, Korea

## Abstract

Using molecular dynamics (MD) simulation methods, we attempted to explain the experimental results on ligand specificity of glucose/galactose-binding protein (GGBP) to β-D-glucose and β-D-galactose. For the simulation, a three-dimensional structure of GGBP was prepared, and homology modeling was performed to generate variant structures of GGBP with mutations at Asp14. Then, docking was carried out to find a reasonable β-D-glucose and β-D-galactose binding conformations with GGBP. Subsequent molecular dynamics simulations of β-D-glucose–GGBP and β-D-galactose–GGBP complexes and estimation of the orientation and stability of water molecules at the binding site revealed how water molecules influence ligand specificity. In our simulation, water molecules mediated interactions of β-D-glucose or β-D-galactose with residue 14 of GGBP. In this mechanism, the Phe16Ala mutant leaves both sugar molecules free to move, and the specific role of water molecules were eliminated, while the wild type, Asp14Asn mutant, and Asp14Glu mutant make hydrogen bond interactions with β-D-glucose more favorable. Our results demonstrate that bound water molecules at the binding site of GGBP are related to localized conformational change, contributing to ligand specificity of GGBP for β-D-glucose over β-D-galactose.

Periplasmic binding proteins (PBPs) exist in the periplasmic space between the inner membrane and outer membrane of gram-negative bacteria^1^. They participate in the high-affinity active-transport system essential to bacterial survival and are important factors of chemotaxis^2^. In X-ray crystallographic studies, the PBP family has been shown to react rapidly with its ligand substrates with high affinity^2^,^3^. Because of this property, PBPs are highly valued for industrial use as components of biosensor systems involving a fluorescent nanosensor^4^,^5^,^6^. In particular, they are studied as glucose-sensing elements that can be used in the development of a blood glucose self-monitoring system^7^,^8^. Glucose/galactose-binding protein (GGBP) is a good example of a PBP that can be used for development of a glucose biosensor protein. GGBP is composed of two domains linked with three loops that serve as a flexible hinge^9^,^10^. The sugar-binding site is located in the cleft between the two domains. In the absence of a sugar, the two domains are far apart and the binding cleft is exposed to solvents. Then, glucose or galactose binding induces drastic conformational changes of the two domains via the hinge loops, and being surrounded by the shifted domains, the binding site is closed to solvents.

PBPs commonly participate in a group-specific transport system in a microorganism^11^,^12^. Therefore, GGBP also recognizes and transports not only glucose but also galactose, with different binding affinity: the dissociation constant (K_d_) of 0.2 and 0.4 μM, respectively^10^. In order to use GGBP as a glucose-monitoring biosensor, this protein should have strict sugar selectivity for glucose with high binding affinity. Nevertheless, at the atomic scale, the sugar selectivity of GGBP has not been explained yet. According to crystal-structure studies, both complexes, glucose–GGBP and galactose–GGBP, share very similar backbone root mean square deviations (RMSDs; under 1 Å) and sugar–GGBP interactions^9^,^10^. Glucose and galactose differ only in stereochemistry at the C_4_ position. The -OH group is oriented to the axial position or equatorial position in glucose and galactose, respectively. Despite the minor difference between glucose and galactose, wild-type GGBP shows distinct stereoselectivity for each sugar.

To turn GGBP into a glucose sensor, many studies based on protein-engineering have been conducted^7^,^8^,^13^, and one of the studies has actually shown that Asp14-mutated GGBPs (as a result of site-directed mutagenesis) have glucose-selective binding affinity under controlled experimental conditions^14^. However, the cause of the glucose-selective affinity in mutated GGBP was not explained. Besides the selectivity issues, several other obstacles to the design of GGBP as a glucose sensor remain. In this respect, identification of the mechanism behind stereoselectivity of GGBP is the most urgent step on the road to the development of a new glucose-sensing system operating under physiological conditions.

In this paper, our goal is to determine how GGBP manifests stereoselectivity to glucose and galactose at the atomic scale. Using computational modeling approaches, we first generated three-dimensional structures of wild-type GGBP and mutant GGBPs and performed molecular dynamics (MD) simulations to describe molecular motions. In the MD simulation results, we observed distinctive hydrogen-bonding patterns within complexes glucose–GGBP and galactose–GGBP and confirmed that the hydrogen bond contributes to stereoselectivity in GGBP. Furthermore, we found that the water molecule in the binding cleft plays a critical role in stereoselectivity of GGBPs.

## Methods

### Preparation of glucose- or galactose-bound GGBP structures and Asp14 or Phe16 mutant structures

Crystal structures of GGBPs bound to β-D-glucose (glucose) or β-D-galactose (galactose) were obtained from the RCSB Protein Data Bank website ( http://www.rcsb.org) (PDB: 2GBP and 1GLG)^9^,^10^. Both raw crystal structures were processed using Protein Preparation Wizard^15^ for use in modeling simulations. The correct bond orders were assigned and hydrogens were added. Hydrogen bonds were optimized based on Epik^16^ calculations for proper pK_a_ values. Water molecules in crystal structures were eliminated except for those within 5 Å of ligands. Added hydrogens were optimized using restrained minimization by means of IMPACT^17^ with the OPLS 2005 force field.

All the mutated GGBPs were constructed based on the prepared crystal GGBP structures. We first eliminated bound sugars for broad searching of residue conformations, and then mutated the target residues and generated various conformations using rotamer search tool. All the generated conformers were minimized using a side chain prediction tool called Prime^18^, and a conformer with the best score was selected as a final structure. Subsequently, we placed ligands into the sugar-binding cleft using a protein-ligand docking application called Glide^18^ and performed minimization on the entire structure using a minimization tool called Impact^17^.

### MD simulations

All MD simulations in this study were conducted using Desmond^19^. The boundary box size was 64.1 × 71.1 × 39.4 Å, and the shape was orthorhombic. MD systems were solvated with TIP3P water model and an ion was added to neutralize the system. NaCl was also added to the solvated system at the concentration of 0.3 M. The NPT ensemble was employed in which the Nose-Hoover thermostat method was set to the reference temperature of 300 K, and the Martyna-Tobias-Klein barostat was set to the reference pressure of 1 bar. Before running the MD simulations, remaining crystal water molecules and hydrogen atoms of glucose/galactose were optimized using Prime minimization tool^18^ and a series of minimizations and short MD simulations were performed to relax the initial MD systems. The Maestro visualization tool was used to analyze the MD simulations.

### Binding free energy calculation

Binding free energy was calculated using the molecular mechanics/generalized born surface area (MM-GB/SA) method of Prime^18^. The Prime MM-GB/SA is typically used to calculate ligand-binding energies and ligand strain energies for a set of ligands and a single receptor. Binding free energy was calculated according to the equation ΔG bind = E_complex − E_ligand − E_receptor. The VSGB2.0 implicit solvation model was utilized, and no flexibility was permitted.

## Results

### Similar binding interactions between galactose-GGBP and glucose-GGBP complexes

**Figure 1:**
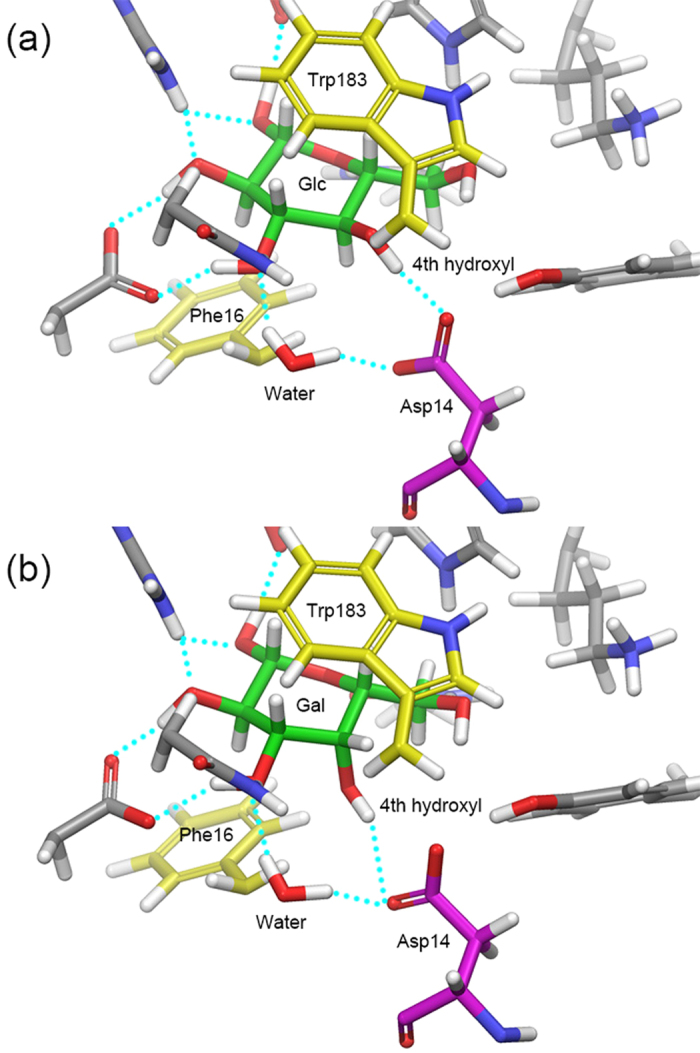
Crystal structures of glucose and galactose bound GGBPs. (**a**) Glucose-GGPB complex, (**b**) galactose-GGBP complex; Cyan-dotted lines indicate hydrogen bonds.

GGBP has stereoselectivity to glucose and galactose with the dissociation constant (K_d_) of 0.2 and 0.4 μM, respectively^14^. To understand this phenomenon at the atomic scale, we first compared two crystal structures of GGBP: in complex with glucose or galactose. According to these crystal structures, GGBP recognizes both glucose and galactose in a very similar way (Fig. 1)^9^,^10^. The pyranose ring of sugars is bound up and down with two aromatic residues (Phe16 and Trp183), and all polar groups of sugars (hydroxyl and ring oxygen) form complicated hydrogen bonds with binding-cleft residues and water molecules. The only observable structural difference is that the epimeric hydroxyl group of sugars at the C_4_ position forms hydrogen bonds with different oxygen atoms of the carboxyl group of Asp14. Nevertheless, the strength of the two hydrogen bonds was expected to be similar because the carboxyl group of aspartic acid is ionized to COO^−^ at neutral pH. In accordance with this observation, binding energies of glucose and galactose calculated for the crystal structures were −72.95 and −72.73 kcal/mol, respectively. The difference in binding energy was under 0.2 kcal/mol, and the binding energy of galactose was slightly more favorable than that of glucose.

### MD simulations of glucose and galactose –GGBP complexes

Stereoselectivity of GGBP did not explain the frozen view of the sugar–GGBP structures. Proteins showed continuous structural fluctuations under physiological conditions, and the water molecule located at the binding site could have various orientations. For these reasons, we utilized an MD simulation to describe the atomic fluctuations of sugars, binding cleft residues, and water molecules. According to a previous study^20^, however, GGBP has a low energy barrier (about 3.5 kcal/mol) between its open and closed conformations, and in the absence of a ligand, GGBP easily crosses the open and closed conformations via thermal fluctuations during MD simulations. We performed four 100 ns test MD simulations in the presence of sugars to evaluate how long GGBP maintains the closed conformation as it is necessary for study of the binding between sugars and GGBP. In our test simulations, closed conformations of GGBP with sugars bound were maintained for about 20 ns (Supplementary information 1). Based on this result, our MD simulations of sugar–GGBP complexes were limited to 20 ns. To measure binding energies of the sugars during the MD simulations, snapshots were extracted at 50-ps intervals, and binding energies were calculated for each snapshot. The average binding energies of glucose and galactose were −50.31 and −42.91 kcal/mol, respectively. During the MD simulations, GGBP showed binding interactions favorable for glucose, and stereoselectivity of GGBP was observed.

**Figure 2:**
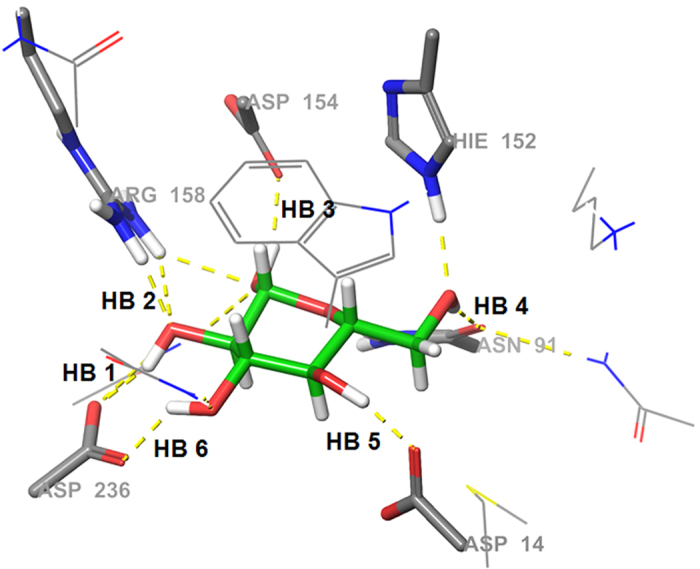
Hydrogen bonds between sugar (glucose) and GGBP.

**Figure 3:**
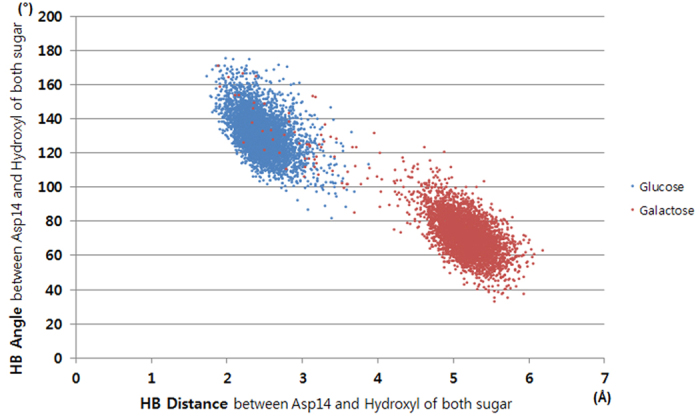
Distance and angle analysis of hydrogen bonds between the fourth hydroxyl of sugars (glucose and galactose) and Asp14 during MD simulations.

**Figure 4:**
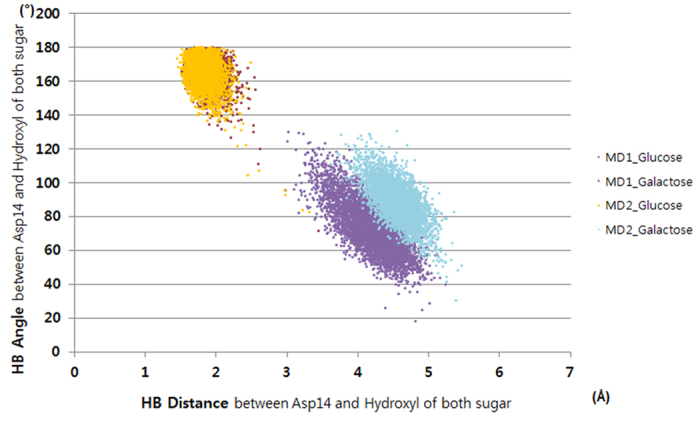
Distance and angle analysis of hydrogen bonds between the fourth hydroxyl of sugars (glucose and galactose) and Asp14 during two additional MD simulations (MD1 and MD2).

To identify the reason for the stereoselectivity during the MD simulations, the sugar–GGBP interactions were analyzed. Because complicated hydrogen bonds have a big contribution to the binding affinity of glucose and galactose for GGBP, we selected six hydrogen bonds in the hydrogen bond network and measured average distances (Fig. 2 and Table 1). Hydrogen bonds No. 1–4 and No. 6 showed distances differing by less than 1 Å depending on binding of glucose or galactose. In contrast, hydrogen bond No. 5 showed average distances of 2.12 and 4.72 Å for glucose and galactose binding, respectively. Hydrogen bond No. 5 forms between Asp14 and the fourth epimeric hydroxyl group of each sugar; this was the only structural difference between glucose- and galactose-bound GGBPs as we mentioned above. For detailed evaluation of the hydrogen bond, the bond distance and angle changes were analyzed (Fig. 3). The optimal distance and angle of the hydrogen bond are approximately 2 Å and 180°, respectively. During the MD simulations, the fourth hydroxyl group of glucose maintained the optimal hydrogen bond distance and angle with Asp14, but the fourth hydroxyl of galactose mostly showed too long a distance and too small an angle with Asp14 to form hydrogen bond. The MD simulations revealed that the hydrogen bond between Asp14 and the fourth hydroxyl group of galactose is weakened, and Asp14 plays a crucial role in GGBP’s distinguishing between glucose and galactose. In an attempt to increase the sampling, instead of elongating the simulation time, we ran simulations twice more under the same setting. The distinctive hydrogen bonds were also observed in both of the simulations (Fig. 4).

### The role of a water molecule in the binding cleft of GGBP

During the MD simulations, both sugars maintained tight binding in the binding cleft via complicated hydrogen bonds and aromatic stacking; therefore, both sugars showed restricted motions (average RMSD was ~ about 0.30 Å). The results showed that the weakness of the hydrogen bond between the fourth hydroxyl group of galactose and Asp14 was induced by localized conformational changes of Asp14.

**Figure 5:**
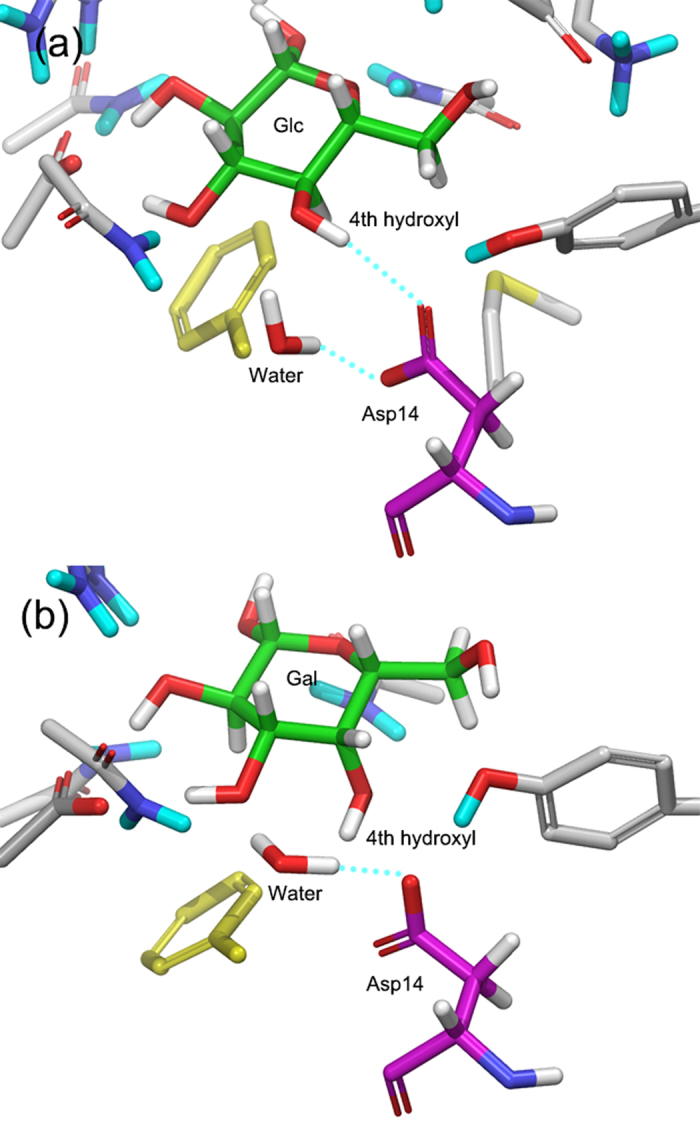
Snapshots of MD simulations. (**a**) Glucose-GGBP complex, (**b**) galactose-GGBP complex; Cyan-dotted lines indicate hydrogen bonds.

One water molecule is located near Asp14 and forms hydrogen bonds with Asp14 (Fig. 1). This water molecule showed distinctive patterns of behavior for the two bound sugars in MD simulations (Fig. 5). In the snapshots of the glucose–GGBP complex, the water molecule forms a hydrogen bond with Asp14, and Asp14 maintains a cooperative hydrogen bond network with not only the water molecule but also the fourth hydroxyl group of glucose: a situation similar to the initial state of the crystal structure. However, in the snapshots of the galactose–GGBP complex, the water molecule moved to the space between the fourth hydroxyl group and Asp14, and Asp14 formed a hydrogen bond only with the water molecule. According to these observations, it was expected that the water molecule would be involved in the structural mechanism of GGBP which determines the sugar selectivity.

**Figure 6:**
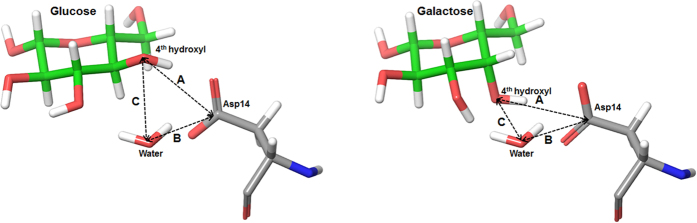
Coordination distances among Asp14, water, and the fourth hydroxyl group of each sugar.

To test this hypothesis, we measured the average coordination distances among Asp14, water, and the fourth hydroxyl group of each sugar (Fig. 6 and Table 2). The distance between Asp14 and the fourth hydroxyl in the glucose–GGBP complex was maintained at the value similar to that of the crystal structure, and the distance between Asp14 and the fourth hydroxyl in the galactose–GGBP complex increased by ~1.8 Å because of localized conformation changes of Asp14. Water molecules in each complex showed distinctive movements. The water molecule in the glucose-GGBP complex moved closer to the fourth hydroxyl group, whereas the water molecule in the galactose complex moved closer between Asp14 and the fourth hydroxyl group. The reason for the different water movements is that the water molecule tends to be located at a spatially optimal site where the van der Waals energy is more favorable. Therefore, in the glucose–GGBP complex, the water molecule moved a little closer to the lower region of the equatorial hydroxyl group of glucose. In the galactose complex, the water molecule moved to the upper region of the axial hydroxyl group of galactose (Fig. 5). The water molecule had a distinctive position in the binding cleft for each bound sugar, and this situation resulted in sugar selectivity by distinctive conformations of Asp14.

**Figure 7:**
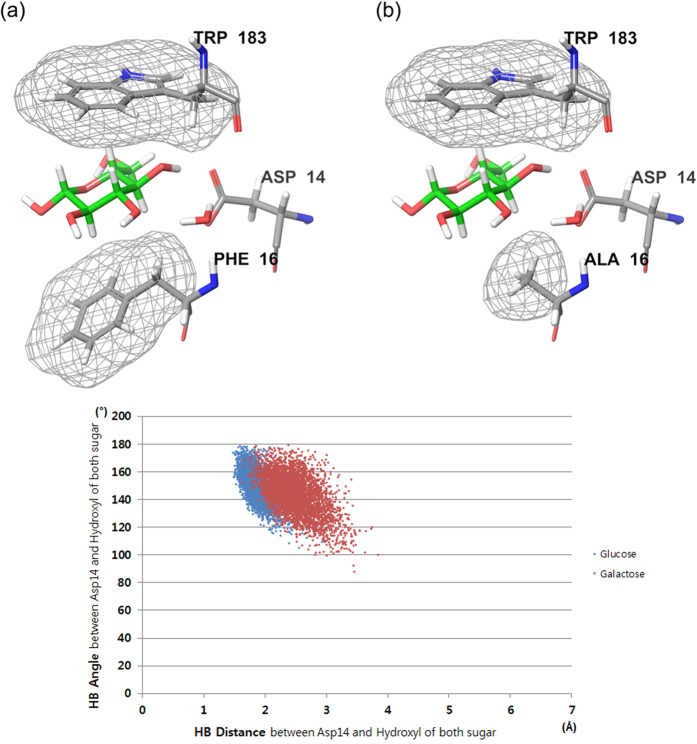
Pyranose ring stacking of GGBP by residues 16 and 183 (top). (**a**) wild GGBP, (**b**) Phe16Ala GGBP; Distance and angle analysis of hydrogen bonds between the fourth hydroxyl of sugars (glucose and galactose) and Asp14 of Phe16Ala GGBP during MD simulations (bottom).

As mentioned above, the pyranose ring of the sugars is stacked with Phe16 and Trp183 on both sides. According to the experimental study, when the Phe16 was mutated to Ala, the binding affinity of both ligands fell to the same level (K_d_ of 2.0 × 10^2^ μM), and the stereoselectivity of GGBP disappeared^14^. We assumed that the role of water in sugar selectivity was nullified in Phe16Ala GGBP. To verify this assumption, we built two Phe16Ala GGBP models (in complex with glucose or galactose) and performed MD simulations. Alanine has a smaller side chain than phenylalanine does. Because of the mutation, additional space where the water molecule can be located was formed at the binding site (Fig. 7). In accordance with our assumption, the water molecule was not anchored and was not involved in the sugar–Asp14 interaction during the MD simulations. Furthermore, a distinctive conformational change of Asp14 did not occur during the binding of galactose to GGBP, and the sugars of both sugar–GGBP complexes maintained equally strong hydrogen bonds with Asp14 (Fig. 7). Although the hydrogen bond patterns were slightly different in the two complexes so that the conformation of Asp14 is more favorable for the glucose to form hydrogen bonds, the difference was too subtle to determine the sugar selectivity. It is only with the distinctive water movements that one can explain the difference in binding affinities of the two complexes.

### The role of water also crucial in Asp14 mutants

In contrast to the Phe16Ala mutation, mutants Asp14Asn and Asp14Glu showed noticeable stereoselectivity—K_d_ of 0.4 and 0.3 μM for glucose, respectively—and no response to galactose^14^. We expected that the water effect would also be strong for both mutations. Therefore, two Asp14 GGBP structures were constructed using the homology modeling approach. Based on the predicted mutant structures, each complex (glucose- or galactose-bound) was constructed using protein-ligand docking. After that, MD simulations were performed under the same conditions as in the prior simulation, and the hydrogen bond between residue 14 and each sugar was analyzed.

**Figure 8:**
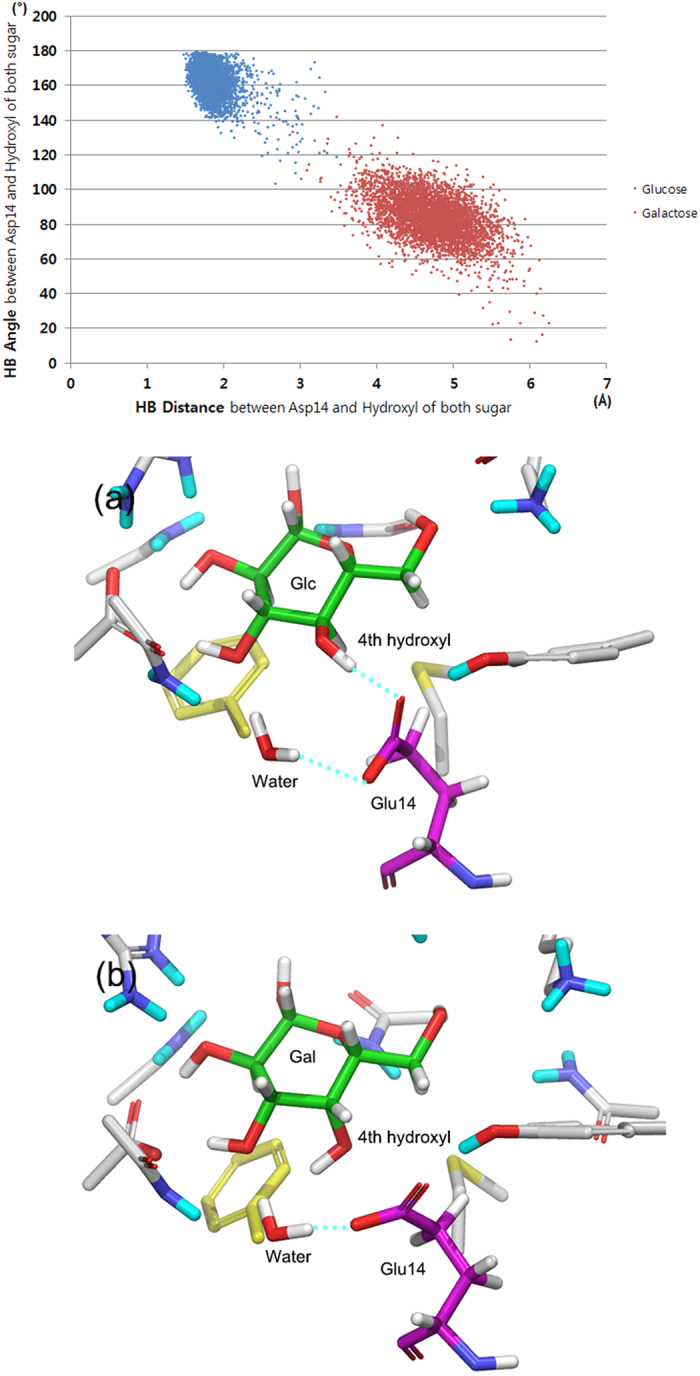
Distance and angle analysis of hydrogen bonds between the fourth hydroxyl of sugars and Glu14 during MD simulations (top). Snapshots of MD simulations (bottom): (**a**) glucose-Asp14Glu GGBP complex, (**b**) galactose-Asp14Glu GGBP complex; Cyan-dotted lines indicate hydrogen bonds.

In both predicted sugar–Asp14Glu GGBP structures, Glu14 forms two hydrogen bonds—with the water molecule and the fourth hydroxyl of the sugars—without a difference between the bound glucose and galactose. Nonetheless, in MD simulations, the hydrogen bond of the glucose–Glu14 GGBP complex remained stable and maintained ideal bond lengths and angles, with the average distance 1.84 Å and angle 162.8°, but the hydrogen bond of galactose–Glu14 GGBP was broken during the MD simulations (Fig. 8).

A similar role of water was also observed in Asp14Glu simulations (Fig. 8). In the case of glucose–Glu14 GGBP complex, the water molecule turned and moved a little from the initial position, and the carboxyl group of Glu14 turned to form a cooperative hydrogen bond network with the water molecule and the fourth hydroxyl group of glucose. In contrast, in the case of the galactose–Glu14 GGBP complex, the water molecule moved to the spatial gap between galactose and Glu14 and pushed out Glu14. Therefore, the hydrogen bond formed only between the water molecule and Glu14, whereas the fourth hydroxyl was excluded from the initial hydrogen network.

**Figure 9:**
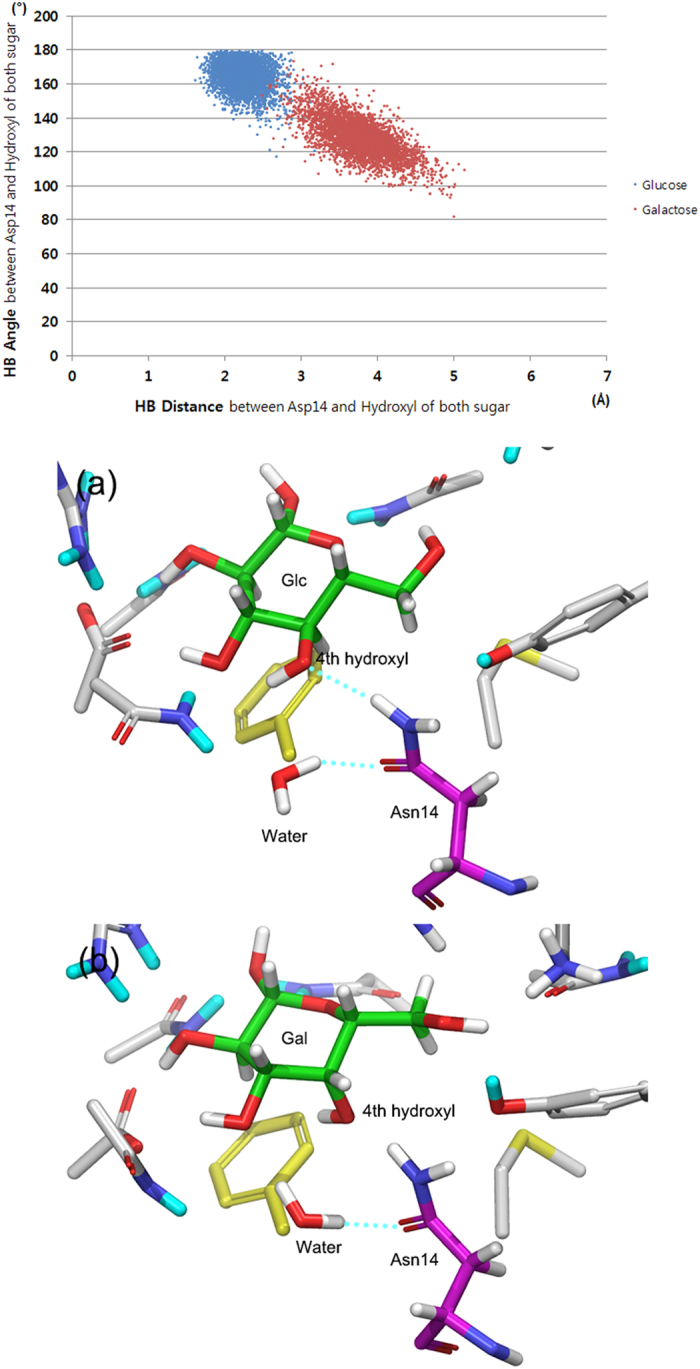
Distance and angle analysis of hydrogen bonds between sugars (glucose and galactose) and Asn14 during MD simulations (top). Snapshots of MD simulations (bottom): (**a**) glucose-Asp14Asn GGBP, (**b**) galactose-Asp14Asn GGBP; Cyan-dotted lines indicate hydrogen bond.

Asparagine has carboxamide in the distal side chain. In the predicted Asp14Asn GGBP, the carboxamide formed two hydrogen bonds—with the fourth hydroxyl and water molecule—and the two sugar–Asn14 GGBP complexes shared similar hydrogen-bond conditions among the fourth hydroxyl, Asn14, and water molecule. In the MD simulations, the hydrogen bond network among the fourth hydroxyl of glucose, Asn14, and water molecule was maintained effectively (Fig. 9). In contrast to this situation, galactose did not form a hydrogen bond with Asn14. Asn14 of the galactose–GGBP complex underwent conformational changes under the influence of the movement of a water molecule and formed a new hydrogen bond with the backbone of Trp183. For the new hydrogen bond, more stable hydrogen-bond patterns than those of galactose–Glu14 GGBP were observed.

### The water-mediated hydrogen bond between GGBP and each sugar

According to the MD simulations of mutated GGBPs, the absence of the hydrogen bond between the sugar and residue 14 prevents the binding of galactose to GGBP. Although wild-type GGBP in complex with galactose showed a hydrogen-bond pattern similar to that of the galactose–Asp14Glu GGBP complex, wild-type GGBP showed the binding affinity (K_d_) of 0.4 μM for galactose unlike Asp14Glu GGBP. In this regard, we expected that the water molecule in wild-type GGBP would form a water-mediated hydrogen bond because the water molecule has a strong potential for hydrogen-bond interactions. Therefore, we analyzed water-mediated hydrogen bonds between each sugar and residue 14 during MD simulations (Table 3). In the simulation of the wild-type GGBP–galactose complex, the frequency of the water-mediated hydrogen bond between Asp14 and the fourth hydroxyl group was 65%. In the simulation of the Asp14Glu GGBP–galactose complex, however, the water molecule maintained a hydrogen bond only with Glu14, and the water-mediated hydrogen bond formed rarely. The difference was caused by one additional methylene group of glutamic acid (in comparison with aspartic acid).

The direct hydrogen-bond interaction was eliminated between residue 14 and the fourth hydroxyl in all galactose-binding GGBPs, whereas only wild-type GGBP showed the water-mediated hydrogen-bond interaction when galactose was bound. The occasional hydrogen bond mediated by a water molecule yields lower binding affinity of wild-type GGBP for galactose.

## Conclusion

In the present work, we demonstrated that a water molecule can play an important role in ligand-protein binding. In the case of GGBP, it was shown that a water molecule mediates GGBP’s binding of glucose and galactose selectively. Previously, the stereoselectivity had not been identified at the atomic scale on the basis of a frozen view of crystal structures. Therefore, an MD simulation was employed here to describe atomic fluctuations of sugar–GGBP complexes, and GGBPs (wild type and mutants) showed more favorable binding energies for glucose; this finding was consistent with experimental data on the binding affinity. During the MD simulations, we found that Asp14 undergoes distinctive conformational changes only in the galactose–GGBP complex, and these changes break the hydrogen bond with the fourth hydroxyl group of galactose. GGBP’s binding affinity was favorable for glucose because of this hydrogen bond. We also found that one water molecule that binds near Asp14 and the fourth hydroxyl group of the sugar is an important regulatory factor. The water effect was also verified in the MD simulations of Phe16Ala, Asp14Glu, and Asp14Asn mutants of GGBP. Our work may help to design new glucose-sensing systems that can be used in diabetes-monitoring sensors. It should also be noted that while experimentally the mechanism of the role of water in GGBP binding may seem to be only a consequence of the conformational structures, computationally considering the explicit interactions with water molecules can give a deeper insight into the mechanism of protein-ligand bindings.

## Additional Information

**How to cite this article**: Kim, M. and Cho, A. E. The role of water molecules in stereoselectivity of glucose/galactose-binding protein. *Sci. Rep.* **6**, 36807; doi: 10.1038/srep36807 (2016).

**Publisher’s note:** Springer Nature remains neutral with regard to jurisdictional claims in published maps and institutional affiliations.

## Supplementary Material

Supplementary Information

## Figures and Tables

**Table 1:** Average distances of six hydrogen bonds between sugars (glucose and galactose) and GGBP during MD simulations.

(Å)	HB 1	HB 2	HB 3	HB 4	HB 5	HB 6
Glucose	2.04	2.77	3.43	2.44	2.12	2.08
Galactose	1.97	2.48	3.01	2.78	4.72	1.82
Δ	0.07	0.29	0.42	0.38	2.60	0.26

(HB1: Asp136-C2 position, HB2: Arg158-C2 position, HB3: Asp154-C1 position, HB4: Asn91-C5 position, HB5: Asp14-C4 position and HB6: Asp236-C3 position. See Fig. 2).

**Table 2:** Average distances between Asp14, water molecule and the fourth hydroxyl of sugars (glucose and galactose) during MD simulations.

	Glucose-GGBP (Å)			Galactose-GGBP (Å)		
	A: Asp14 - 4^th^ hydroxyl of glucose	B: Asp14 - Water	C: Water - 4^th^ hydroxyl of glucose	A: Asp14 - 4^th^ hydroxyl of galactose	B: Asp14 - Water	C: Water - 4^th^ hydroxyl of galactose
**Initial state (crystal structure)**	3.53	3.67	3.74	3.41	3.91	3.84
**MD simulation**	3.47	4.02	3.28	5.25	3.16	3.28
**(−)**	−0.06	+0.35	−0.46	+1.84	−0.75	−0.56

See Fig. 6.

**Table 3:** Frequencies of water-mediated hydrogen bonds between the fourth hydroxyl of sugars (glucose and galactose) and residue 14 during MD simulations.

	4th hydroxyl - water (A)	water - residue 14 (B)	Water-mediated hydrogen bond(A∩B)
wild GGBP - glucose	99%	99%	99%
wild GGBP - galactose	65%	65%	65%
Asp14glu GGBP - galactose	7%	94%	7%
