# Supplementary material for: The role of water molecules in stereoselectivity of glucose/galactose-binding protein
Source: Sci Rep. 2016 Nov 9;6:36807. doi: 10.1038/srep36807 (PMC5101532; doi:10.1038/srep36807)
Supplement: Supplementary Information [file srep36807-s1.pdf]

Supplementary information

The role of water molecules in stereoselectivity of glucose/galactose-binding protein

Minsup Kim and Art E. Cho*

Supplementary information 1

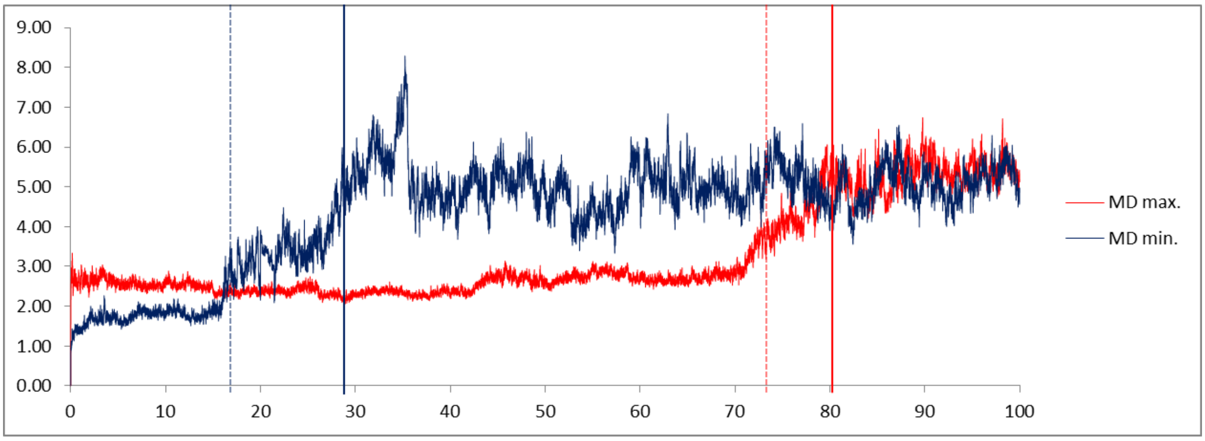

We prepared four distinct MD systems from a single GGBP-sugar complex with different orientations of solvents and ions. During 100 ns of simulation time, GGBPs in all of the four differently prepared MD systems underwent conformational changes to be in the open form. Starting times of conformational changes vary, however. The fastest (Blue line; MD min.) started to change at 18 ns and changed completely at 29 ns and the slowest (Red line; MD max.) started to change at 73 ns and changed completely at 80 ns. The energy barrier between the open and closed conformations of apo structure is rather low at about 3.5 kcal/mol and in the case of the complex, the conformational change starts when water molecules start attacking inter-domain interactions of binding site and hinge loops[1]. In our test simulations, GGBP kept its closed conformations at least 20ns in all different MD settings.

1. Unione, L. *et al.* Unraveling the Conformational Landscape of Ligand Binding to Glucose/Galactose-Binding Protein by Paramagnetic NMR and MD Simulations. *ACS chemical biology* **11**, 2149-2157, doi:10.1021/acschembio.6b00148 (2016).
